# “What is the score?” A review of football-based public mental health interventions

**DOI:** 10.1108/JPMH-03-2017-0011

**Published:** 2017-12-18

**Authors:** Bettina Friedrich, Oliver John Mason

**Affiliations:** 1 Research Department for Clinical, Educational and Health Psychology, University College London, London, UK; 2 School of Psychology, University of Surrey, Guildford, UK

**Keywords:** Mental health, Inclusion, Evaluation, Football, Soccer, Physical activity

## Abstract

**Purpose:**

Football exercise as an intervention for people with severe mental health problems has seen an increasing interest in the past years. To date, there is, however, no comprehensive review of the empirical evidence regarding the effectiveness of these interventions. In this review, the authors have comprised the research findings from the peer-review literature as well as the theoretical approaches to football exercise as an adjunct treatment. This overview will be informative to everybody who is planning to develop a football intervention for this population as well as to the people who are preparing evaluation studies that measure the effectiveness of such interventions. The paper aims to discuss these issues.

**Design/methodology/approach:**

The authors identified research papers in the peer-review literature that feature empirical findings on “football interventions” that aim at improving mental and/or physical well-being in participants with mental health problems. The authors are using the term “football intervention” here in the sense that the participants actively took part in football exercise, so the authors excluded studies in which the participants only watched football or used football as a metaphor to discuss mental health problems. In a table, the authors indicate the definition of the target group, targeted outcomes, measured outcomes, form and frequency of the intervention as well as the research method(s).

**Findings:**

The authors identified 16 studies on 15 projects. The majority of studies were qualitative and had positive findings in which the participants reported increased well-being and connectedness, elevation of symptoms and improved physical well-being. The outcomes of the quantitative studies, however, were mixed with some results suggesting that not all intended goals were achieved. There seems to be a need for more quantitative studies to triangulate the qualitative findings. Interestingly, most interventions take place in the UK. Many studies fail to give detailed methodological information and often the aims of the interventions are vague or not stated at all.

**Research limitations/implications:**

Due to the heterogeneity of the studies and relative scarcity of evaluation projects on football interventions for people with mental health problems, the authors could not conduct an in-depth systematic review. Furthermore, the information on methods was often unsatisfying and despite efforts to get more detailed input from the authors of cited papers, those gaps could not always be filled. Instead of coming up with a crystal-clear summary of whether and how football interventions work for everybody, topics were identified that need to be addressed in the planning of interventions, in evaluation studies, in implementation efforts and in the theoretical discourse.

**Practical implications:**

This paper constitutes a helpful overview for everybody who is interested in the theoretical background of football interventions for people with mental health problems, for people who are planning to develop respective interventions, for researchers who engage in evaluation projects that look into the effectiveness of football interventions (or similar exercise interventions) as well as for the people who are interested in how football interventions can be implemented. This paper is likely to make a contribution to the advancement of alternative exercise interventions that aim at improving mental, physical and social health in people with mental health problems.

**Social implications:**

This paper will help putting the topic of football interventions (and similar, alternative exercise interventions) further up on the public health agenda by providing an overview of the empirical evidence at hand and by specifying advantages of the approach as well as pointing out actions that need to be taken to make football a recognised, evidence based and viable option for adjunct mental health treatment that is attractive to potential participants as well as funders as well as to the potential participants.

**Originality/value:**

There is no comprehensive summary to date that provides a (reasonably) systematic overview of empirical findings for football interventions for people with MH problems. Furthermore, the literature on the theoretical background of these interventions has been somewhat patchy and heterogonous. This paper aims at filling both these gaps and identifies the issues that need to be covered in the planning of respective interventions and evaluations. This paper will be useful to everybody who is developing football interventions (or similar alternative adjunct exercise interventions), who is conducting evaluation research in this area and who is interested in the implementation of football interventions.

With the increasing prevalence of mental health problems and thus growing burden on health systems (Mayor of London, 2014; McCrone *et al.*, 2008), there is an increasing appetite for mental health interventions which complement “standard” or conventional treatment. In particular, “recovery-based” interventions outside mainstream psychiatric services that focus on the strengthening of people’s abilities, gaining hope and social reconnection and empowerment are getting rising attention (Le Boutillier *et al.*, 2015; Slade, 2009). Recovery interventions often focus on the principles of fostering inclusion and enhancing self-esteem through social activities and can be seen as a critical counter reaction to treatments that are strongly linked with the medical model of mental illness which advances biological treatments. In addition to this, the medical model is often seen as strongly deficit-oriented whereas approaches whose roots are often attributed to positive psychology usually focus more on the strengths in patients and ways of enhancing them (Seligman and Csikszentmihalyi, 2014).

A prominent group of recovery-focussed mental health interventions are based on sport or exercise activity, and some empirical evidence supports its effectiveness. Mood disorder is the most studied with Stathopoulou *et al.* (2006) concluding from meta-analysis of efficacy studies that “exercise can be a powerful intervention for clinical depression” (p. 188), leading to the suggestion that clinicians “consider adding exercise interventions to their armamentarium to strategies to help patients in distress” (p. 190). Schuch *et al.* (2016) have recently provided a systematic review of the impact of exercise interventions for people with depression. Exercise as an adjunctive mental health intervention has also been studied, albeit less frequently in aiding anxiety (see, e.g. Asmundson *et al.*, 2013; Jayakody *et al.*, 2013; Anderson and Shivakumar, 2015), self-esteem (Barton *et al.*, 2012), schizophrenia (Faulkner and Sparkes, 1999; Carter-Morris and Faulkner, 2003; Campbell and Foxcroft, 2003) as well as general mood (Barton *et al.*, 2012; Powers *et al.*, 2015). Mechanisms of clinical change have also been the focus of qualitative enquiry such as the narratives of three men with schizophrenia engaging in physical activity (Carless and Sparkes (2008). Mason and Holt (2012a) reviewed the qualitative literature to identify the mechanisms in which participants with mental health problems benefit from mental health interventions and described a range of physical, psychological and social mechanisms that may explain the impact of exercise interventions for people with mental health problems.

Interventions aimed at improving the physical health in people with mental health problems are not only important in terms of supporting psychosocial recovery, but also to address the particular physical health needs in this population with cardiovascular vulnerabilities high on the list. Osborn (2001), for example, has argued that since morbidity and mortality rates are high in people with psychiatric problems, programmes aiming at improving the physical health are crucial though hitherto far too neglected.

Football, or “soccer” in some cultures, has gained a lot of attention in terms of being a popular tool for exercise interventions for people with mental health problems (for reviews, see Spandler and Mckeown, 2012; Darongkamas *et al.*, 2011). There are several reasons for its popularity beyond its obvious appeal as a mass participation sport engaging one in five adults in England. We now explore some of the benefits for physical health, mental and social health triggered by football as an intervention, and why it may be well suited to some under-reached parts of the population.

## What makes football a suitable intervention for people with mental health problems?

### “Match fit?” Physical health

Oja *et al.* (2015) conducted a systematic review in which they compared the outcomes of different sports disciplines with regard to physical health benefits. The authors found that football was one of the disciplines that are most beneficial to the participants in terms of physical health improvements, including aerobic fitness, cardiovascular function, metabolic fitness and running performance. Krustrup *et al.* (2013, 2014) have also provided empirical evidence for the valuable impact of football training with regard to cardiovascular functions, especially in untrained men (Krustrup *et al.*, 2009). Given the fact that in particular cardiovascular functions are often impaired in people with mental health problems (Batelaan *et al.*, 2016; Scott *et al.*, 2016), football seems a very suitable form of exercise intervention in this population.

### “Getting a kick out of it”: mental health

Battaglia *et al.* (2013), among others, argue that, in addition to the physical benefits of exercise, many psycho-educational and social characteristics of football practice are highly relevant to participants’ lives: “[…] it is important to acknowledge the positive effects derived from the inner features of team activities, such as social and motivational opportunities, when soccer is employed to increase mental health; examples of these effects include having opportunities of social interaction, cooperating with others, respecting rules, acquiring other skills suitable for everyday life, sharing leisure experience, experiencing personal achievement, being part of a winning team, and receiving encouragement of other people with the same disorder” (p. 596).

Nielsen *et al.* (2014) used self-determination theory (Ryan and Deci, 2000; Deci and Ryan, 1985) to explain why participants are “intrinsically motivated” to participate in football exercise. According to the theory, people have an intrinsic need for autonomy, competence and relatedness and they will feel intrinsically motivated to partake in activities that fulfil these needs. Nielsen *et al.* (2014) argue that football as a team sport is more suitable to fulfil these essential needs and that it is therefore that participants feel more rewarded, arguing: “[…] the higher frequency of social interactions in the football activity creates more positive perceptions of social relations which in turn increase intrinsic motivation and ultimately adherence” (p. 73). Seime and Vickers (2006) similarly emphasise the importance of football’s potential to trigger intrinsic motivation to exercise in people with depression; noting that finding the motivation and drive to do sports activity can be a hurdle for this population. Extending the traditional conception of what sport may offer in “Just a Game? The Therapeutic Potential of Football”, Steckley (2005) explored the possibilities of using football as therapeutic intervention for young people in residential care. She suggested that football provides structure, contributes to the development of pro-social values and behaviour and can instil a sense of mastery and self-value in participants. Furthermore, recent research has shown that football can also be beneficial to cognitive functioning in the participants. Alesi showed that children who participate in football training had increased spatial organisation (Alesi *et al.*, 2015) as well as increased agility, visuo-spatial working memory, attention, planning and inhibition skills (Alesi *et al.*, 2016).

### “A place on the team”: social inclusion

Football has the clear potential to increase mental well-being by fostering interaction with others and training or rehabilitating social skills, enabling social inclusion. The effects on individual well-being are hard to disentangle from these social effects as these are interdependent to a large degree. Many people with (severe) mental health problems experience isolation (Brophy and Harvey, 2011) for a range of reasons: they drop out of the labour market (Perkins and Rinaldi, 2002); experience breakdown of meaningful social relationships; and also feel isolated as a consequence of experiencing mental health-related stigma and discrimination (Hinshaw, 2009; Link *et al.*, 2001) and/or self-stigma (Link *et al.*, 2001; Corrigan and Watson, 2002). Public health interventions are well placed to tackle several of these consequences with socially highly valued activities such as football prominent among them. For individuals often solely identified by their mental health problems whereby they are very devalued by society at large (e.g. as a “schizophrenic”), the potential for football to offer a degree of perceived social status and normalcy is considerable.

Darongkamas *et al.* (2011, p. 20) make it clear that perceived inclusion is a fundamental part of the effect of football interventions on mental well-being: “[…] benefits to participants’ mental health and social and family life were the result of, or mediated by, meeting other people in a similar situation. This may have had the effect of increasing a sense of belonging, community and acceptance, which are all principles of social inclusion”. In an ethnographic study of a community-based team of service users, Mynard *et al.* (2009) underline the importance of the intervention’s ability to meet the patients’ psychosocial needs. Entitled using Liverpool supporters’ famous chant “You will never walk alone […]”, McKeown *et al.*’s (2015) study in the North West of England explores football’s quality to unify “in acts of solidarity and togetherness”. Some argue that football has the power to bring people together from different backgrounds: “Significantly, it is the ability of football to ‘reach’ so many people that makes it a true game for all, irrespective of gender, age, ethnicity, religious belief, disability, socio-economic or health status” (Parnell and Richardson, 2014, p. 823).

## The attraction of football to young men

It is a particular asset of football and football-based interventions that it is popular among a demographic which is often considered “hard to reach” in the context of mental health interventions: young men across a range of social, class and ethnic backgrounds (Mellor, 2008; Parnell and Richardson, 2014). Despite the high prevalence of mental health issues in young men, they remain reluctant to engage with statutory mental health services (Prior, 1999; Oliver *et al.*, 2005). The fact therefore that football is an attractive activity and “hook” for this demographic makes is an interesting tool for engagement. In their analysis of the effectiveness of the “It’s a Goal” intervention, Pringle, Zwolinsky, McKenna, Robertson, Daly-Smith, and White (2014), for example, point out the importance of this quality to football and how it makes it an important platform from which to reach out to men and encourage them to speak about and deal with mental health issues. Various high-profile professional footballers both with and without their own mental health issues have helped establish this platform in the media space, and more local initiatives have capitalised on it: “It’s a Goal”, for example, uses football metaphors to encourage men to open up and to speak about mental health problems and is an noteworthy example of how football as a “theme” can become of interest to men (Pringle, 2004; Spandler *et al.*, 2013). According to Pringle (2004), it is not just the playing of football that can be a meaningful intervention in mental healthcare, but watching football or using football stadiums to host interventions can be similarly useful.

Robertson *et al.* (2013) also emphasise the fact that football is attractive for young men and make the important points that this bears the potential to be used intentionally as a means to engage them in positive health activities; the authors point out that rugby, for example, has been used as intervention for the same reason (Witty and White, 2011).

However, male role stereotyping, or “machismo”, within football also has its downside as (Spandler *et al.*, 2013) among others have pointed out. According to these authors, football and engaging with football overly emphasises the gender rules which might be counterproductive to therapeutic goals in the mental health intervention. This can create “paradoxical spaces” (Spandler and Mckeown, 2012) – while football is supposed to help men with opening up and with increase self-esteem, it runs the risk of replicating stereotypes and gender inequalities that play a negative role in men’s health and social outcomes. Striving for dominance and experiencing respective performance pressure are, for example, such issues; the authors point out that football can be competitive and that it is “literally a game of winners and losers, in which one team succeeds at the others expense” (p. 141). This competitive side can potentially be counterproductive in the attempt to use football as a mental health intervention: “It is easy to see that the rules of football (emphasizing action, strength and competition) may conflict with the rules of therapy (favouring emotions, expression of vulnerability and concern)” (Spandler *et al.*, 2014, p. 149). The potential effects of a more or less competitive atmosphere in such interventions depend of course on the personal preferences of the individual intervention participants. While the competitive element can be the main point of attraction to some as it provides them with an opportunity to prove themselves, it can be intimidating to others and can be considered as an unnecessary pressure by those who just want to “kick the ball” and who are more after an opportunity to socialise and get some physical exercise. There does not seem to be a “one size fits all” solution and it is important to bear these differences in individual preferences in mind when designing and implementing football interventions. Where possible, it might be worthy forming groups that feature different level of competitiveness and assign participants according to their personal preferences. Both male role stereotyping and competitiveness clearly need to be explicitly addressed within any football-based public health intervention to enable therapeutic possibilities for recovery. On a similar note, the level of physical exercise suitable for the participants has to be carefully considered. Given the increased prevalence of cardiovascular health issues in people with mental health problems, respective issues could be triggered or worsened by intense workout, so facilitators should be mindful of these issues and – where necessary – should monitor cardiovascular health in participants.

Despite these issues, there is an array of arguments that speak in favour of the suitability of football for an exercise-based adjunct public health intervention as outlined above.

## Empirical evidence

What empirical evidence to date has examined the effectiveness of football interventions in a mental health context? Some years ago, Pringle (2009) suggested that “the challenge for those involved in football and mental health work is to begin to develop a body of evidence that stands up to scrutiny when commissioners and service planners suggest that “this is all a bit of a jolly really” and that staff should “get on with their real work”. The main objective of this review is to provide an overview of the current empirical evidence regarding the benefits of football interventions for those with mental health problems.

Table I shows an overview of published studies identified by systematic search. We explored the available literature using the search engines Web of Science and Google Scholar. We entered search terms “(Football OR Soccer) AND (Mental disorder OR depress* OR psychiatr*)”. We included literature which describes football as an active intervention (i.e. actively played by the participants); for people who have mental health problems; that included empirical outcomes from quantitative and/or qualitative evaluation studies with any outcomes relevant to physical, mental and/or social health. We also used the bibliographies of all relevant literature to identify any further evaluation studies. In addition to this, we searched online more widely for “football interventions” to identify any relevant projects that described evaluation work that might be relevant. We contacted several national mental health organisations in Europe, Australia and North America to identify any further football intervention evaluations, though no additional reports were identified. Reference to one study was provided by a reviewer of this paper. In total, 16 relevant studies were identified in the present overview.

One study reported on a scheme involving several sporting activities of which football was the majority activity (Butterly *et al.*, 2006). It is impossible to rule out other studies in which football is included but not explicitly described.

Unsurprisingly perhaps for such a diverse area of study, reports in the literature varied widely in the degree of description of the aims of evaluation, and the nature of the interventions. In constructing this overview, we attempted to identify, extract and summarise the most important information on the studies (see Table I).

The studies vary quite widely according to the participants targeted, the nature of the intervention and the methodologies employed. In terms of participants, some exclusively aim at those with severe and enduring mental health problems, with one occurring wholly in a rehabilitation context. Others are much more diverse in terms of mental health difficulties, even, in the case of “Imagine Your Goals”, including around a third without mental health problems – something not explicitly envisaged at the outset of this very substantial programme in 16 professional clubs. Some schemes are exclusively male, while others have varying proportions of females. Some aim at younger participants, even with a particular “youth” focus, while others extend across the lifespan. In terms of setting, the most common was a football club, with both local and professional clubs represented. Many involved connections to participants via local mental health charities, statutory health and residential services.

Though it is somewhat difficult to be specific about the aims the commonest were increasing social contact/inclusion (seven schemes); improving physical health/fitness (six) and improving self-confidence (six). A minority of evaluations used quantitative indices with the Warwick-Edinburgh Mental Well-Being Scale being the most popular. The majority utilised a range of qualitative methods differing in their level of formality of data gathering and analytic methods (at least in the level of detail given).

The number of participants included in the evaluation studies varied widely. For example, Hynes (2008) conducted qualitative interviews with only three participants, while Henderson *et al.* (2014) had follow-up data on as many as 157 participants in their quantitative study. On a similar note, inclusion criteria of the interventions (and hence the respective evaluation) differed markedly. While Steckley’s (2005) observation included boys from in residential care with emotional and behavioural problems, Grant and Oldknow (2008), for example, conducted an analysis on an intervention that include both male and female adult players with severe and enduring mental health problems. See Table I for details across the range of studies.

Due to the diversity of studies (aims, target groups, measures and outcomes) it is probably not appropriate to condense the findings into a single narrative: however, the empirical evidence thus far is universally encouraging. The majority of studies containing qualitative analysis have a high degree of consistency of thematic content. These themes can often be grouped into elevated self-esteem/empowerment/independence, emotional well-being/pleasure/enjoyment, the experience of inclusions/belonging/connecting and improvement of physical well-being. Rather than narrowly targeting a single outcome, almost all report significant impacts on psychological, social and physical health. Quantitative data on the other hand is relatively scarce, and failed to reach significance level on a number of measures (see e.g. Henderson *et al.*, 2014), though typically sample size and measure completion rates are, perhaps unsurprisingly, rather low. As most of these studies do not have a control group, it cannot be ruled out that some of the observed effects in the quantitative studies might be due to the concurrent therapy when football intervention was used as an adjunct treatment form. While Wood *et al.* (2005) point out that a lack of control group is common in rehabilitation studies, it would of course be desirable if future quantitative evaluations would use control groups to disentangle the effects of primary and adjunct treatment where feasible. In the meantime, qualitative studies should help better understanding the mechanisms behind impact of football interventions.

Interestingly the clear majority of these studies have been conducted in England, with two in Scotland and one in Australia. This might be due in part to the particular role football and football clubs play in the culture and everyday life in England. It would certainly be interesting to obtain more empirical evidence from similar projects in other countries in order to establish in how far popularity and impact of similar football interventions are comparable to the findings gathered in England.

## Discussion and outlook

The synopsis of empirical evidence while supportive of football-based adjunct mental health interventions is far from conclusive due to the wide range of interventions and methods used in studies. As a minimum football seems to be a feasible and acceptable tool to many participants, particularly young men, with the aim of improving physical, mental and social well-being particularly suited to a public health context outside of a clinical setting where it has largely been employed to date.

In line with the recovery-based principles, interventions are not symptom- or deficit-focussed, instead aiming to improve overall quality of life of people experiencing mental health issues – tackling diverse challenges including social inclusion, stigma, physical health and feelings of empowerment, for example, that have the potential to make an immense difference for this group. In addition to the direct outcomes for the participants, it is at least plausible that mental health-related stigma can be decreased by showcasing the presence and abilities of people with mental health problems in the community through football exercise, tournaments and leagues. Furthermore, when these football events also involve members from the general public, these interventions can be seen as a type of social contact intervention that are a common tool in fostering inclusion as well as decreasing stigma (Evans-Lacko *et al.*, 2013): football seems to have a very unifying character and indeed global popularity across a very wide range of ethnic communities. These characteristics may make it a suitable vehicle in different countries and ethnically diverse urban areas where increasing inclusion is paramount (Nathan *et al.*, 2010).

Football, however, should not be seen as a “wonder drug” that can be expected to work universally and under all circumstances. Instead of using a one-size-fits-all approach, it is desirable for interventions to clearly define the target group, to assess abilities and needs of potential participants and to tailor form, intensity and frequency of the football intervention accordingly. One important issue, for example, is whether competitiveness or collaboration is desirable. While a sense of achievement might be engendered by stressing performance, success and defeating others this can be at the expense of greater anxiety, fear of failure and encouraging collaboration (see e.g. Steckley, 2005). Furthermore, gender issues can be a potential area of conflict (Spandler *et al.*, 2014). Such issues need to be addressed in the planning stage and monitored during the course of interventions for those projects that involve both men and women. In general, it seems important to identify, address and monitor potential conflict issues. Especially when working with very vulnerable target groups, reasonable supervision and reliable monitoring (emotional and physical health status, group issues) would seem to be essential ingredients of a well-planned intervention, not just for evaluation purposes but in particular to prevent possible harm for the participants by the intervention. Lastly, and as importantly, defining the exact intended outcomes of the intervention (on both, a group and individual level) appears imperative. While this might seem fairly obvious, several studies omit details of the intervention or of intended outcomes. Defining clear aims does not only seem important for tailoring evaluations appropriately, but also to clarify what participants can expect perhaps giving them the opportunity to personalise their own goals in terms of fitness, participation or achievement off the pitch (e.g. exit routes into employment, training or voluntary work).

It is important to emphasise at this point that football interventions are seen by all studies as an adjunct rather than a replacement for formal mental health treatment. In the best possible scenario such interventions can help increase emotional well-being to the point that the participants might experience a reduced need for mental health treatment, particularly of an acute or involuntary nature. However, it would be naïve to assume that playing football can directly replace formal mental health treatment and/or need for medication, especially in groups with severe mental health problems.

So what needs to be done to help football, or indeed any physical exercise, to be recognised as a promising adjunct intervention? After reviewing the literature over a decade ago, Callaghan (2004) recognised that “exercise improves mental health and well-being, reduces depression and anxiety and enhances cognitive functioning. Although exercise seems to improve the quality of life of those living with mental health problems, its value is seldom recognized by mainstream mental health services” (p. 482). It is in the public health arena that football and other physical activity/exercise interventions are increasingly been developed, albeit often disconnected from statutory service provision, regulation and evaluation processes.

In order to be properly recognised as an effective adjunct treatment form in an era of evidence-based practice, it is imperative to have more – and more specific – empirical evidence with regard to the impact of these interventions. From a health policy perspective, this also includes studies that investigate their cost-effectiveness and uniqueness of contribution. Due to the potentially positive effects of football interventions (and other similar physical activity interventions for this matter) on mental, physical and social health, it is likely that an implementation of more adjunct treatments of this kind might prevent some of the financial burden caused by mental health problems (and related issues) on the welfare system and the medical services. More detailed information on the extent of the reduction of these extremely high economic health costs that might be achieved through such interventions would be very valuable to obtain in detailed cost-effectiveness analyses. Such data would be an asset in persuading policymakers and other stakeholders to support the development, implementation and further evaluation of football interventions. Furthermore, empirical evidence might also encourage further collaborations between football clubs and associations like FIFA and FA, for example, with intervention facilitators and researchers. The growing sense of social responsibility by football clubs (especially in England) can be used for the advantage of such interventions (Henderson *et al.*, 2014).

Empirical evidence, however, is not just essential for persuading potential stakeholders, but – when done comprehensively – can elucidate the key ingredients for success. This might help in tailoring interventions and making them more effective in general, and for specific target groups. A very informative and insightful overview of what some experienced experts consider indispensable in football interventions and respective evaluations is offered by Pringle, Hargreaves, Lozano, McKenna and Zwolinsky (2014). Given the multiple targets of change, it is important that evaluation combines quantitative indices (e.g. physical health indicators, psychological scales) with qualitative approaches (e.g. interviews, focus groups, participatory observation, ethnography). Social desirability can be of bigger concern in qualitative approaches (Choy, 2014) and there is potentially a Hawthorne effect at play here (Sedgwick and Greenwood, 2015; Roethlisberger and Dickson, 2003) which might be stronger in the collection of data from interviews and focus groups than of survey data, for example. Outcome-based evaluation is a necessity here.

### Summary

In summary, there is a growing evidence base that is broadly supportive of football-based public health interventions aimed at improving emotional, physical and social well-being in people with mental health problems. At present, this is largely qualitative and provides quite a detailed picture as to the perceived health and social benefits both by participants, health staff and other stakeholders. The majority reported positive outcomes in terms of self-esteem, social inclusion and emotional/physical well-being. It is very unclear, however, which interventions work, for whom they offer most benefit, and what are the most significant and enduring positive outcomes. All interventions operated in the non-profit or charity sector with some possessing links of varying formality with statutory health and social service providers. There is a clear need for greater quantitative evidence for this area of public health so as to gain a solid foundation with potential funders and other stakeholders. Health economic evaluation was notable by its absence and is also a major gap requiring attention for more widespread and enduring service development in this area.

## Figures and Tables

**Table I:** Football-based intervention studies in mental health

Evaluation	Project name/description	Form/frequency	Target group	Intended outcomes	Methods	Identified themes
O’Kane and McKenna (2002)	Five-a-side football project, Lanarkshire, Scotland	Five-a-side tournament Winter league (teams playing 24 games) Summer league	Clients with chronic and enduring mental health problems	Improving levels of physical fitness Enhancing physical self-concept Enhancing levels of self-confidence and self-esteem Encouraging improved communication skills Reducing barriers and promote better staff/client relations Increasing sociability	Interviews with clients and carers (qualitative)	Self-reported increase of general well-being Level of fitness Increased levels of self-confidence, communication easier Clients felt more motivated and considered participation in other similar physically active sports Carers perceived their family members as more proactive and communicative, more alert and in touch with their surroundings
Hynes (2008)	“Positive Mental Attitude” (PMA) League, England (London-wide)	Training 1-2× per week; matches played on monthly basis	Men and women who are experiencing or recovering from mental illness	“Improving lives” Overcoming stigma and exclusion Developing positive attitude Educating players on the importance of healthy eating Improving general fitness, weight loss, smoking cessation Increasing confidence and self-esteem Learning about importance of team work	Interviews of three participants (anecdotal evidence)	Some anecdotal evidence for elevated symptoms. Participants reported gaining independence, confidence, self-belief, happiness and motivation. Employment. Enjoyment, equilibrated mood, regaining of structure, inclusion
Darongkamas *et al.* (2011)	Park House United Football Club, Staffordshire, England	Voluntary training at football club	Men with mental health issues	(No aims specified)	Quantitative: Warwick-Edinburgh Mental Well-Being Scale Resource Generator UK Qualitative interviews of ten participants	Improvement in mental health, attitudes about themselves, general well-being Five themes: social inclusion, changes in attitude and behaviour, enjoyment and importance of the club, changes to mental health, self-confidence
Henderson *et al.* (2014)	“Imagine Your Goals” (Sixteen Premiership clubs, England)	Regular training sessions	Men and women with mental health problems	Improving physical health, building confidence, reducing social isolation Social contact, bringing people with and without MH problems together (Campaigning for Time to Change)	Warwick-Edinburgh Mental Well-Being Scale, The Resource Generator-UK (RG-UK) Focus groups	Improvement only at personal skills subscale, individual skills (The Resource Generator-UK; RG-UK) Improved levels of health and fitness, including weight loss; social contact/inclusion; improved well-being; improved self-esteem, creating more structure, new experiences, appreciation of exit routes, fear of project ending
Carter-Morris and Faulkner (2003)	“Longfield United”, Birmingham, England	Regular meetings in team and regular tournaments (international)	Male service users with enduring mental health problems (three out of five had schizophrenia)	Positively impacting on the lives of service users Inclusion/ getting participants out of social isolation	Semi-structured interview of ten participants Qualitative analysis: themes analysed through inductive analysis	Three themes: Promoting normalisation and a personally meaningful opportunity for social interaction Challenge auditory hallucinations and delusional beliefs characteristic of schizophrenia Side effects of medication identified as key barrier to greater participation
McElroy *et al.* (2008)	Manchester’s “Grassroots Initiatives” (England)	Care Standards Improvement Programme (CSIP) league; 15 minute matches, six-a-side version of the game	“Socially excluded, particularly those who experience mental health/learning-related difficulties”	Heath improvement Internal experience Inclusion Confidence/security	Likert scales on experience of energy, optimism, anxiety, development of coping skills, sense of inclusion of 131 participants	Perceived physical and mental health improvements Reduction of anxiety Perceived improvement of coping Improved tolerance towards others Subjective feeling of inclusion Increased confidence
Mynard *et al.* (2009)	“RecLink” league, Melbourne, Australia	A community-based Australian Rules Football league	16-60-year-old men with psychiatric, and cognitive impairments (and others)	Tackling social, occupational disadvantages associated with mental health	Ethnography (one team observed through one season, five in-depth interviews)	Three major themes associated with the experience of belonging to this RecLink football team: Spirit of inclusion Team building Meaning of team involvement
Carless and Douglas (2008)	Football (and other exercise) at Rehabilitation Centre in Leeds, England	Voluntary Football Training at Rehabilitation Centre	Male Residents with serious mental illness issues at a Rehabilitation Centre	Recovery/inclusion	Participant observation Medical records Interviews	Reconnecting to old self Gaining satisfaction and sense of achievement Social network Emotional well-being Time use/keeping busy Improvement of social and interpersonal difficulties Attitudes towards others
Carless and Sparkes (2008)					Qualitative approach: semi-structured interview (3 participants)	Feeling supported Self-confidence Happiness/satisfaction/enthusiasm Fitness
Mason and Holt (2012b)	“Coping Through Football”, London, England	Two training sessions per week with Leyton Orient coaches; occasional workshops and match visits	Younger men with severe mental health problems	Improving mental and physical well-being Reducing social isolation through inclusion and help finding exit routes into volunteering and/or employment	Interviews of participants, coaches and referrers for grounded theory analysis	Identifying with past self Service with a difference Opening up to the social world Psychological Safety “Feeling good” Empowerment
Grant and Oldknow (2008)	“Recreational Enterprise Assisted Client Training”; Football Training Academy, Doncaster, England	40-week basic training programme	Men and women with severe enduring mental health problems, along with individual levels of social dysfunction	Development of football skills Health promotion Healthy lifestyle Healthy eating First aid and photography skills	Qualitative Interviews (details of evaluation method and number of participants not stated)	Improved football skills Physical fitness Better self-image Higher self-confidence Feeling part of a team Common identity Improved moods Exit routes/new opportunities
Brawn *et al.* (2015)	Mental health and football well-being league, North West of England	Ten six-a-side local mental health teams. In total, nine months of a year	Men with mental health problems	Widening the social opportunities Facilitating improvement in their mental and physical health	Interviews of seven participants aged between 25-63. Narrative-oriented inquiry – themes identified	Self-reported improvement of physical and psychological well-being and sense of self Sense of belonging Connecting to previous history Distraction from current problems Sense of achievement
Steckley (2005)	League for young people with emotional and behavioural difficulties, Scotland	Participation in league	Boys in residential care with emotional and behavioural difficulties	Promoting pro-social values Enhancing Resiliency Providing healing Enhancing environments Self-experience	Observation/case studies	Enjoyment of training/game Empowerment Stronger sense of self Resilience Inclusion
Butterly *et al.* (2006)	Community-based physical activity project (MUSCSEL) in Leeds, England	Weekly five-a-side soccer sessions overseen by staff from Leeds United Football Club	Male and female mental health service users in the Leeds area	Increasing the physical activity levels of mental health service users in the Leeds area This should result in reduced demand not only on social services but also on NHS resources allocated to mental health provision	Questionnaire Semi-structured interview	Enjoyed exercise/activity but barriers prevented them from participating further b) – Increased self-confidence Increased energy level Reduction in the incidence of severity of their symptoms Felt more confident socially and made more friends Exit routes: some participants planned to go for more educational and vocational qualifications felt fitter weight loss positively affecting self-image and self-esteem
Magee *et al.* (2015)	Three football projects in the UK (anonymous)	Recreational/competitive sessions Healthcare Treatment Programme Support Workshops Programme	Male participants were recruited though drug and alcohol services, youth offending and probation, mental health services	Assisting mental health recovery: service user engagement; stigma; and social isolation	Semi-structured interviews with participants, project staff and associated professional medical staff and partner agency staff (thematic analysis)	Connecting to people who experience similar mental health issues/sense of community Sense of safety, social connectedness, and peer support Counteracting stigma Some participants criticised idea of “recovery model” as they felt it implied “something needs fixing”
Abib *et al.* (2010)	Football intervention of the Psychosocial Care Centres (PSCCs)/Porto Alegre	Football workshop weekly for one-and-a-half hour	Adults with mental health problems (mainly from PSCC)	Psychosocial rehabilitation of people with mental health problems, reintegration into community	Ethnography methodology	Integration Self-control proactive role in receiving MH care opportunity for self-organisation
